# Modifying Older Adults' Daily Sedentary Behaviour Using an Asset-based Solution: Views from Older Adults

**DOI:** 10.3934/publichealth.2016.3.542

**Published:** 2016-08-09

**Authors:** Calum F Leask, Marlene Sandlund, Dawn A Skelton, Emmanuelle Tulle, Sebastien FM Chastin

**Affiliations:** 1 Glasgow Caledonian University, School of Health and Life Sciences, Institute of Applied Health Research, Glasgow, UK; 2 Umea University, Department of Community Medicine and Rehabilitation, Umea, Sweden; 3 Glasgow School for Business and Society, Department of Social Sciences, Media and Journalism Glasgow, UK

**Keywords:** sitting, older adults, qualitative, physical activity, intervention

## Abstract

**Objective:**

There is a growing public health focus on the promotion of successful and active ageing. Interventions to reduce sedentary behaviour (SB) in older adults are feasible and are improved by tailoring to individuals' context and circumstances. SB is ubiquitous; therefore part of the tailoring process is to ensure individuals' daily sedentary routine can be modified. The aim of this study was to understand the views of older adults and identify important considerations when creating a solution to modify daily sedentary patterns.

**Method:**

This was a qualitative research study. Fifteen older adult volunteers (mean age = 78 years) participated in 1 of 4 focus groups to identify solutions to modify daily sedentary routine. Two researchers conducted the focus groups whilst a third took detailed fieldnotes on a flipchart to member check the findings. Data were recorded and analysed thematically.

**Results:**

Participants wanted a solution with a range of options which could be tailored to individual needs and circumstances. The strategy suggested was to use the activities of daily routine and reasons why individuals already naturally interrupting their SB, collectively framed as assets. These assets were categorised into 5 sub-themes: physical assets (eg. standing up to reduce stiffness); psychological assets (eg. standing up to reduce feelings of guilt); interpersonal assets (eg. standing up to answer the phone); knowledge assets (eg. standing up due to knowing the benefits of breaking SB) and activities of daily living assets (eg. standing up to get a drink).

**Conclusion:**

This study provides important considerations from older adults' perspectives to modify their daily sedentary patterns. The assets identified by participants could be used to co-create a tailored intervention with older adults to reduce SB, which may increase effectiveness and adherence.

## 1. Introduction

There is a growing public health focus on the promotion of successful and active ageing [1]. Recently, there is an increasing body of evidence identifying sedentary behaviour (SB, defined as any seated or reclined posture with a MET of < 1.5 [2]), as detrimental to both physical [3] and mental [4] wellbeing. This has created an additional cost for healthcare services, as SB is a risk factor of all-cause mortality [5] and in Canada, for example, reducing sitting time could potentially decrease spending on heart disease, cancer, hypertension and diabetes by a cumulative $ 2.6 billion in the next 25 years [6]. The deleterious health outcomes of prolonged SB may occur even if an individual is physically active [7] and as a result, several countries, such as the United Kingdom, have introduced specific guidelines that recommend SB should be reduced where possible [8]. Of all segments of society, older adults occupy the most time in SB [9], spending an average of 8.5 hours a day seated [10].

To reduce this public health cost, there is a clear need for interventions to decrease sedentary time in older adults. Previous work has shown that these interventions are feasible [11] and can be effective in decreasing total sedentary time in the short-term [12]. A key element in the short-term success of these interventions was adopting an individualised approach to goal setting and feedback; which were based on set behaviour change strategies.

Tailored interventions, which Rimer and Kreuter [13] define as strategies that can be implemented specifically to the needs of an individual, have been shown to significantly outperform generic health behaviour change interventions [14], in addition to being effective in physical activity studies [15]. A review conducted by the European Joint Programme Initiative DEDIPAC (Determinants of Diet and Physical Activity) Knowledge Hub [16] highlighted that future intervention development needs to be informed by qualitative work to tailor solutions to individuals and their settings.

There have been two recently published qualitative studies which explored the determinants of SB in older adults in different countries and settings [17],[18]. There were multiple barriers to reducing SB reported by older adults, ranging from personal factors (such as pain from standing up) to environmental factors (such as a lack of park benches to allow for activity pacing) [17],[18]. Additionally, authors showed that in future interventions to reduce sedentary time, it is crucial that they are tailored to individuals' daily routine. This is due to SB being ubiquitous [19]; therefore interventions must act throughout the day and adapt to the changing circumstances of the daily routine. In addition, in both studies, older adults expressed views that they would not adopt interventions requiring significant disruptions of daily routine [17],[18]. Therefore, a solution needs to be developed which fits into individuals' daily life.

The aim of this study was to identify important considerations when creating a tailored solution to reduce sedentary time that fit in older adults' daily routine from their perspective.

## 2. Materials and Methods

### 2.1. Study Design

In this qualitative research study, focus groups were conducted with older adults in 4 different community settings to identify considerations when creating a solution to modify individuals' daily sedentary routine. Ethical approval was granted from the Glasgow Caledonian University School of Health and Life Sciences Ethics Committee.

### 2.2. Sample

A convenience sample of 15 community-dwelling older adults, aged > 60 years, were recruited over a 4 week period. A total of 4 focus groups were conducted, with 1 focus group occurring in each of the following distinct settings: a retired person's association (6 participants), a university gym class (3 participants), a Scottish country dancing group (3 participants) and an ethnic minority social group (3 participants). Pre-existing groups were chosen to increase the cohesiveness of participation [20]. Demographic information was compiled using questionnaires prior to each focus group. Collected data included: age, gender, Scottish Index of Multiple Deprivation (SIMD) and sedentary time was assessed using a domain specific questionnaire: Longitudinal Aging Study Amsterdam (LASA) [21]. The SIMD is the Scottish Government's official tool for measuring socio-economic distribution throughout post-code areas in Scotland, with scores being ranked from 1 (most deprived areas) to 5 (least deprived areas) [22]. LASA is a valid and reliable self-report questionnaire to assess the average amount of time older adults spend in various sedentary activities, including reading, screen-time and transport [21]. Written consent was provided prior to participation.

### 2.3. Protocol

Focus groups were based on semi-structured interviews and discussions were founded on a series of exploratory questions regarding how to modify older adults' daily sedentary patterns. Discussions were initiated using a topic guide (supplementary material), which included incentivisation, consciousness of SB and tools to monitor SB. Examples of questions asked included: *“can you think of any reasons why you would get up out of your chair?”* and *“what do you think you could do to get yourself up off the chair more at home?”* Focus groups took place in locations where older adults participated in their normal social groups, for example participants who attended the university gym class attended their focus group on the university campus. Three researchers (CL, TS and RL) were present for each focus group, with two facilitating discussion and another taking detailed notes on flipchart paper as a reference point for member checking at the end of the focus group [23] and for use during data analysis [24]. All discussions were audio recorded.

### 2.4. Data Analysis

The audio discussions from each focus group were transcribed and analysed thematically [25]. Thematic analysis was chosen as a widely established [26] and tested method [27] which allows for patterns of experience to be recorded [28], for example understanding older adults' experiences of their daily sedentary routine. The analysis followed a multi-phase coding process: initial familiarisation with the data; creating initial codes; searching for themes amongst these codes; reviewing the themes; naming and defining the themes; and presenting the final report [26]. Data were analysed independently by 2 researchers (CL and RL) and their findings cross-referenced as a method of verifying the data [29], with disagreements being resolved by consulting a third researcher (SC).

## 3. Results

### 3.1. Participants

Focus group 1 had 6 participants (1 male, average age = 81 years) with an average sedentary time of 13.1 hours and average SIMD score of 3. Focus group 2 had 3 participants (2 male, average age = 77 years) with average sedentary time of 9 hours and average SIMD score of 3. Focus group 3 had 3 participants (1 male, average age = 66 years) with an average sedentary time of 14.3 hours and average SIMD score of 4.3. Focus group 4 had 3 participants (0 male, average age = 74 years) with an average SIMD score of 3.7 and due to issues with translation, sedentary time was not reported.

### 3.2. Themes

Three themes emerged from the analysis: (1) *solutions should be tailored to the individual* due to the ineffectiveness of generalised solutions; (2) the *resources of daily living* could be used as a solution, including physical and psychological resources; (3) *solutions should exclude technology* due their inconvenience and being problematic (Table 1).

**Table 1.** Themes and sub-themes derived from focus groups.

Theme	Sub-theme
Solutions should be tailored to the individual	Generalised solutions ineffective
	Complex individual differences
Use resources of daily living	Physical assets
	Psychological assets
	Interpersonal assets
	Knowledge assets
	Activity of daily living assets
Solutions should exclude technology	Technological devices problematic
	Inconvenience

#### 3.2.1. Solutions Should be Tailored to the Individual

**Generalised solutions ineffective** — It was commonly discussed by participants that any solution must be based on individuals' circumstances. One commented that a generalised solution would not be effective and a personal element was crucial to gaining her attention: *“If you knew that there was a problem, so it wasn't just a generalisation that's really more specific to the person, that's what I would be looking for, to be tailored just to me” (Participant 11, P11).* Taking ownership of their own health appeared to be the reason for this rationale. Individuals' expressed that they were in charge of managing their health in a positive way: *“I think it's up to me because my health, my responsibility” (P7)*. Ownership would influence the speed at which they carried out their daily living: *“I just find that I want to do everything at my own tempo” (P13).*

**Complex individual differences** — In addition to expressing the view that solutions should be tailored to their daily routine, older adults also voiced the necessity for solutions to be adapted to the complex and very different situations and settings they find themselves in. Within this group, age was cited as a strong influence on individuals' daily routines: *“All those here today, we're all different ages and we've all got different things to do” (P14)*. As a result, it appeared that a variety of solutions would be necessary to meet the preferences of a large range of people. The possibility of a health message as a solution was explored, with one commenting that individuals' physical health status may influence whether this would be an effective or ineffective tool to use: *“I don't think it would necessarily help me but it might help some people who have particular problems, like you say, cholesterol, or blood pressure or what have you” (P12)*.

#### 3.2.2. Using the Resources of Daily Routine

It was identified that a simple solution to interrupt sedentary patterns could be to use what an individual already does to break their sitting. For example, the activities that people do to interrupt sedentary periods along with the reasons they break their SB were acknowledged as resources which could be harnessed and tailored on individuals' circumstances: *“But I think honestly, I've got enough resources here to make myself move, now that I've recognised the fact that I'm sitting long” (P8).* These resources were framed as “Assets” and 5 sub-themes emerged: physical assets, psychological assets, interpersonal assets, knowledge assets and activity of daily living assets.

***Physical assets*** — Participants reported that the effect of prolonged sitting on their body provided a strong natural incentive to stand up regularly. From this, two clear assets could be identified. The first and most commonly mentioned was ‘reducing stiffness’, which participants' experienced while sitting for too long: *“I think my knees begin to stiffen up. You know. We go to church on a Sunday, you sit for an hour*” *(P7)*. Secondly, ‘reducing soreness’ was identified as an asset that encouraged them to stand up to relieve the unpleasant physical sensation: *“If you sit long, you get lazy and joint pain. When you get up, you feel better” (P11).* Individuals' health could fluctuate on a daily basis; with most participants suggesting these daily changes were important in how often sitting could be broken during the day. From this, “feeling energetic” was identified as an asset which could be commonly used on “good” days to stand up more frequently. One commented: *“well some days the spirit moves me and I'll clean the house”(P15)* whilst another took the opportunity, when feeling good, to complete a lot of daily tasks *“because the next day you might think I'm not too well today, so I've not got that [daily tasks] to do” (P14).* Participants perceived there to be additional negative consequences on their physical health if they sat for too long. One stated that *“sitting can sometimes impede circulation and that's bad … as we all know” (P10)*, suggesting that “improving circulation” was an asset this individual possessed to interrupt long sedentary periods.

***Psychological assets*** — Psychological markers were also identified as influences on sitting. Some participants suggested that depending on the sedentary activity, they would interrupt this to do something deemed more important. This asset was identified as “guilt of sitting”: *“I think guilt comes into it as well. You can be watching a film and say ‘look I really shouldn't have done this I ought … not to be watching that’” (P10)*. This closely related to the value that individuals placed on specific sedentary activities. Television was often referred to as a SB with low value, meaning participants would spend limited time sitting in this activity before interrupting it. This asset was identified as “value of seated activity”: *“the television is so duff, I watch the news and that's it” (P5).*

***Interpersonal assets*** — Numerous participants suggested that their family were a positive influence on interrupting their sitting time, identifying “family support” as an asset. One commented that when she had her *“son and his family up … I never sat down because they were all around the house moving about” (P4).* Family also facilitated breaking sitting time by enabling older adults to use another asset: “stand up to answer the phone”: *“I actually don't sit and answer the phone and make a phone call” (P7).* Participants' friendship groups provided an additional form of social support. One particularly active individual who still regularly attended the gym identified “friend support” as an asset he regularly uses, as he *“surrounded myself with active people, who would rather go for walk than go for a pint” (P9).* The final asset identified in this theme was “pet responsibility”, which several participants noted, for example being required to exercise their pet regularly: *“when I see people out walking their dog, then I think that's a good thing to have” (P4).*

***Knowledge assets*** — In this sub-theme, participants noted that not all older adults knew how to interrupt SB. Therefore, “education of breaking sitting” was identified as an asset which could be used: *“I must stand more, therefore what am I going to do … I haven't been trained to think about this” (P10).* Another asset that older adults could harness was having “increased awareness of sitting”, with one commenting that monitoring her sitting would act as a trigger to modify her sedentary patterns more frequently: *“I think it'll [monitoring] make us more conscious of not to be sitting too long” (P16).* The final asset in this sub-theme was “knowing benefits of breaking sitting”, as several participants were not aware that this would benefit their health: *“If you know the benefits of health you ensure you have good health … it's a mind-set I think” (P10).*

***Activity of daily living assets*** — Incidental activities, which were integral components of daily life, were all spoken about as positive influences on reducing sitting time. From this, a number of assets were identified and commonly associated with domestic tasks. Examples of these assets included preparing meals: *“obviously to make the tea” (P16)*; “using the bathroom”: *“your bladder could be the reason (to break sitting time)” (P1)* and “taking medication”*: “I have to take tablets. When you're on tablets, you know, they're quite often” (P1)*. Other daily tasks which were also identified as assets were: “getting a drink”: *“especially at night time, watching TV and I realise I haven't moved since an hour” (P7)* and “housework”, which most of the women cited they did during the day: *“don't like to sit during the day because we like to finish our housework” (P12)*. Housework was not an asset reported by any men, however one discussed that “DIY (do it yourself)” would interrupt sedentary time: *“if there's anything to be done in the house, a repair or a room to be painted, or the windows to be cleaned” (P9).*

#### 3.2.3. Solutions Should Exclude Technology

When exploring how any potential solution should be presented, participants generally felt that any solution which uses technology would not be desirable. There were two main reasons for this which emerged from the data, including 1) previous problematic experiences with technological devices and 2) some devices are regarded as inconvenient and may result in unwanted attention from participants' peers.

***Technological devices problematic*** — Despite many using mobile phones and tablets, participants were in agreement that they would not prefer a technological solution. One spoke of having problems previously when attempting to monitor her activity which acted as a deterrent from future use: *“When I've used a pedometer, I've never had success with it. I bend over and knock it off” (P6).* What participants appeared to be more receptive to was keeping a tangible record of their activities using a pen and paper as they felt it would be more meaningful: *“Keep a diary. That's one thing that's physical that you have to write down” (P10).*

***Inconvenience*** — Discussion brought about the possibility of having a wrist worn device to monitor sedentary time, however participants were worried this may result in undesired attention from their peers: *“people would say that's strange, what have you got around your wrist … it might attract unwanted comments … why are you wearing that? What are you doing? Why are you doing it?” (P10).* Participants were shown examples of technological devices which could be used to monitor SB and, whilst most regularly used technology, they generally did not see a benefit of using this to monitor their sitting: *“I don't really know if it would make any difference to my life wearing one of these” (P14).*

## 4. Discussion

Within the focus groups, older adults identified that they wanted to use the things they already do as part of their daily routine and things that already trigger them to stand up. These activities and reasons to stand up were collectively named as “Assets” that can be used as a solution to modify daily sedentary patterns. There are several perspectives that can be drawn on to understand why older adults recommended such an approach. One theoretical perspective which may explain these views and experiences is salutogenesis [30], which suggests that individuals already possess the positive traits necessary to enhance desirable health behaviours, for example modifying their daily sedentary patterns. Salutogenic theory proposes that individuals possess generalised resistance resources (GRR's) to enhance desirable health traits, which can include but are not be limited to: physical, emotional and interpersonal-relational resources [30]. Here, three of the asset categories which older adults identified, mimic the GRR's proposed by Antonovsky [28]. The categories identified by the older adults in this study were “Physical assets” (relating to salutogenic physical assets, such as improving circulation), “Psychological assets” (relating to salutogenic emotional assets, such as valuing seated periods) and “Interpersonal assets” (relating to salutogenic interpersonal-relational assets, such as family support). Salutogenesis hypothesises that these GRR's are effective to enhance health as they are coherent with individuals' life, due to their meaningfulness, comprehensibility and manageability [30]. Despite being advocated as an approach to use in health promotion [30], it has been largely overlooked in the literature, yet may provide an effective framework to enhance health at the individual level.

The asset-based solution identified by participants may also be investigated from a health promotion perspective. Whilst utilising assets is still a relatively novel concept at the individual level, this notion has been previously examined on a community level [31]. These community level assets were derived from material resources, including economic, natural and technological capital [32] and were utilised to build the capacity for health promotion [31]. Personal level assets were used by each individual to build their own health improvement capacity [31], which is appropriate due to the heterogeneity of this population and as such, individualised sedentary patterns. This reinforces the justification of participants in this study advocating an asset-based approach for being an effective solution to successfully modify daily sedentary routines. Future work should aim to collaboratively develop an intervention with older adults and incorporate these assets to evaluate its effectiveness.

The concept of assets can also be explored by comparison with the COM-B model [33]. The model suggests that the sources of a behaviour can come from individuals' capacity (either physical or psychological), opportunity (social or physical) and their motivation (automatic or reflective) [33]. The assets identified by participants in the focus groups resonate closely with the capacity and opportunity constructs identified by Michie et al [33]. For example, individuals' had the physical capacity to break sitting when they wanted to reduce stiffness, but also had the psychological capacity to stand up when they did not value a certain SB. Additionally, the opportunity to interrupt SB could be manifested in a social form, for example standing up to answer the door, or a physical form, such as taking medication. This highlights that several assets are dependent on the context of SB [34] and reinforces the benefit of providing a range of assets which can be used at different times of the day for different reasons.

This study does have some limitations. Individuals who volunteer for research projects may have different sedentary patterns compared to the general population; therefore the assets identified here may not be representative of other older adults. However, participants did also attempt to identify some assets which other older adults not associated with the research project, may have used. Also, as this sample was mostly older old adults (mean age = 78 years), this may also explain why certain themes emerged, such as solutions should exclude technology, that may not necessarily be representative of younger older adults' views. In addition, the findings may have been influenced by bias, social desirability and greater input from more outspoken and confident participants, although the researchers did endeavour to ensure that each individual had a chance to speak. While the participants were from very diverse backgrounds, they did not constitute a fully representative sample of the diversity found in the wider older adult population. Therefore, the actual list of assets might not be entirely exhaustive and their definition applicable in all settings. However, the asset model as a solution for modifying sedentary periods could be applied widely and this personalised and sensitive approach may be an effective way to address gender, age and ethnicity differences.

The deleterious health effects of too long spent in SB has resulted in an additional cost for healthcare services [35] and several countries have released explicit guidelines to reduce prolonged sedentary periods where possible [8]. There is a clear requirement for interventions to reduce SB and due to being SB occurring periodically throughout the day [19], a major component of tailoring future interventions of this nature is to modify the daily routine [17],[18]. It is interesting to note that older adults' total sitting time naturally changes from day to day and can sometimes fluctuate by up to 4.5 hours per day [36], which is a larger variance than any previous work implementing a traditional behaviour change theory model. Therefore, it could be suggested that individuals already possess an inherent capacity to change [17] and do so regularly throughout the day. Therefore, incorporating an approach which can modify the daily routine may be a valuable strategy to adopt. A similar strategy has successfully been used previously for balance and strengthening exercises in older adults [37], reinforcing the feasibility and benefit of utilising this approach.

## 5. Conclusion

This study provides several considerations, voiced by older people, when creating an interventional solution to modify older adults' daily sedentary patterns. One consideration is to use the resources an individual already possesses to interrupt SB, collectively framed as “Assets”. Encouraging older adults to change their SB based on these incidental disruptions of their daily routine may be a useful tool to incorporate into future interventions to increase effectiveness and adherence. In addition, these may be tailored based on individual needs and circumstances and older adults felt the use of technology was not necessary or desirable. Researchers and practitioners should work in collaboration with older adults to co-create a tailored intervention which effectively embeds these considerations which can then be distributed to a larger group of older adults to assess its effectiveness.

## Focus Group Topic Guide

1. Obtain information - outstanding paperwork & consent forms. 2. Introduction

- Thank everyone for coming, appreciate input.
- Hope to hear from everyone, so please let everyone speak, one at a time.
- Please be honest and candid & ask if our question is unclear
- Any questions take longer to answer & not directly related- leave till end
- We will start by introducing ourselves and then we will ask you to introduce yourself, using the name you would like to known as.

**Topic: general discussion around sitting** We are investigating the amount of time over 65 year olds spend sitting at home. This can include things like sitting watching TV, eating meals, doing crafts, chatting on the phone, resting etc. We are **not** talking about physical activity- we all know exercise is good for us & many studies exist. The topic today is about the time you spend sitting down which you may or may not have ever thought about. 1. Have you ever thought consciously about how much time you spend sitting? 2. What would be the main things that you do when sitting? 3. a. Do you think that the amount of time you spend sitting changes from day to day? b. How does your sitting time change from day to day? c. What about seasonally? d. Can you think anything other than the weather that causes you to sit more or less from day to day e. Can you think of any reasons why you might change the amount of time you spend sitting within a day. 4. Do you feel different in the times when you sit more compared to those when you sit less? 5. Can you think of any reasons why you would get up out of your chair? **Topic: general discussion around breaking sitting** Please can I ask you all to stand up for a moment. Researchers have found evidence to show that breaking up the length of time you spend sitting can improve your health and increase you life span. These beneficial effects are not related to exercise- they are a separate issue. What we are saying is, whether you exercise or not- if you can break up the time you spend sitting, by standing up for even a couple of minutes- just like this, or going to make a cup of tea, can help you stay healthy and live longer! You may all sit down now, thank you. 6. Is this something you were already aware of? 7. If you were to help a friend (of around the same age) to get up off the chair more, then what would you say to them? 8. a. What do you think you could do to get yourself up off the chair more at home? b. What time of the day do you think it would suit you to get up more, and why? 9. If you could have ANY type of help to get you off your chair more often, what would that be? **Topic: general discussion around social influences** 10. a. We know that having other people or a person to support you can help. Do you think this would help you reduce the time you spend sitting? b. Who would you like this person/people to be? Prompts; Younger/same age/relative/professional? 11. What type of support? Prompt- Could this be a text message, face to face, email, phone call. 12. What is the driving force behind why you would want this person to support you? Prompts; eg; advice, competitive, sharing information, nagging? (i.e. what kind of relationship would they be to you, prompt; mentor, friends, family) **Topic: discussion around incentives** 13. Would it help you to have some incentive/reward/gift to reduce your sitting time? 14. What incentive (reward/gift) might help you to do this? 15. How regularly would you like this incentive? **Topic: discussion around tools & feedback** 16. Research has shown us how important it is to accurately record the amount time of you spend sitting and how many times you stand up. Can you think of any method to do this? How would you like to know your progress? **Topic: show & explain examples and bodyworn devices** Here are some examples of what we could use; Note: Pedometer (can be attached to your hip or worn around your neck on a lanyard and can read a simple number, for example the length of time you've been sitting for). Wrist Worn Monitor (number can show how long you've spent sitting, light display up the top may represent trying to stand up as many times as you can, goes green when you hit your goal). Phone (receive a text message telling you how long you've been sitting for or how many times you've got out of your chair in a day). Could you see yourself using any of these? Why not? What would you prefer? 17. What type of information would you like this device to give? Prompts- total sitting time in hours, number of times you sat per day? 18. How long would you like to monitor this for; hours, 1 day, few days, constantly? 19. Monitoring with a device can inform you how well you did that day, by telling you how you improved your healthy behaviour (of reduced sitting). Do you think this information/feedback will help you? 20. How would you like to receive this informative advice (feedback) of how much you improved? eg figures, graph, oral, motivational speech? 21. How often would you like this information (feedback)? **Topic: discussion around salutogenesis;** You are all healthy. Your here and well. From this stand point, why do you strive to be healthy? What makes you different from someone else who is less healthy? **Finally member check whiteboard and thank participants**

## References

[1] Hallal PC, Andersen LB, Bull FC (2012). Global physical activity levels: surveillance progress, pitfalls, and prospects. Lancet.

[2] Yates T, Wilmot EG, Davies MJ (2011). Sedentary behavior: What's in a definition?. Am J Prev Med.

[3] Henson J, Yates T, Biddle SJH (2013). Associations of objectively measured sedentary behaviour and physical activity with markers of cardiometabolic health. Diabetologia.

[4] Teychenne M, Ball K, Salmon J (2010). Sedentary behavior and depression among adults: A review. Int J Behav Med.

[5] Van der Ploeg HP, Chey T, Korda RJ (2012). Sitting time and all-cause mortality risk in 222 497 Australian adults. Arch Intern Med.

[6] The Conference Board of Canada (2014). The economic impact of reducing physical inactivity and sedentary behaviour.

[7] Dogra S, Stathokostas L (2012). Sedentary behavior and physical activity are independent predictors of successful aging in middle-aged and older adults. J Aging Res.

[8] Department of Health (2011). Start active, stay active: a report on physical activity from the four home countries' Chief Medical Officers.

[9] Matthews CE, Chen KY, Freedson PS (2008). Amount of time spent in sedentary behaviors in the United States, 2003–2004. Am J Epidemiol.

[10] Harvey JA, Chastin SFM, Skelton DA (2013). Prevalence of sedentary behavior in older adults: a systematic review. Int J Environ Res Public Health.

[11] Gardiner PA, Eakin EG, Healy GN (2011). Feasibility of reducing older adults' sedentary time. Am J Prev Med.

[12] Fitzsimons CF, Kirk A, Baker G (2013). Using an individualised consultation and activPAL™ feedback to reduce sedentary time in older Scottish adults: Results of a feasibility and pilot study. Prev Med.

[13] Rimer BK, Kreuter MW (2006). Advancing Tailored Health Communication: A Persuasion and Message Effects Perspective. J Commun.

[14] Noar SM, Benac CN, Harris MS (2007). Does tailoring matter? Meta-analytic review of tailored print health behavior change interventions. Psychol Bull.

[15] Krebs P, Prochaska JO, Rossi JS (2010). A meta-analysis of computer-tailored interventions for health behavior change. Prev Med.

[16] Chastin SFM, Buck C, Freiberger E (2015). Systematic literature review of determinants of sedentary behaviour in older adults: a DEDIPAC study. Int J Behav Nutr Phys Act.

[17] Chastin SFM, Fitzpatrick N, Andrews M (2014). Determinants of sedentary behavior, motivation, barriers and strategies to reduce sitting time in older women: a qualitative investigation. Int J Environ Res Public Health.

[18] Greenwood-Hickman MA, Renz A, Rosenberg DE (2016). Motivators and Barriers to Reducing Sedentary Behavior Among Overweight and Obese Older Adults. Gerontologist.

[19] Sartini C, Wannamethee SG, Iliffe S (2015). Diurnal patterns of objectively measured physical activity and sedentary behaviour in older men. BMC Public Health.

[20] Onwuegbuzie AJ, Dickinson WB, Leech NL (2009). Toward more rigor in focus group research: A new framework for collecting and analyzing focus group data. Int J Qual Method.

[21] Visser M, Koster A (2013). Development of a questionnaire to assess sedentary time in older persons - a comparative study using accelerometry. BMC Geriatr.

[22] The Scottish Government (2012). Scottish Index of Multiple Deprivation 2012: A National Statistics Publication for Scotland 18 December 2012.

[23] Carlson JA (2010). Avoiding Traps in Member Checking. Qual Rep.

[24] Kitzinger J, Barbour R (1999). Developing Focus Group Research: Politics, Theory and Practice.

[25] Holliday A (2006). Doing and writing qualitative research.

[26] Braun V, Clarke V (2006). Using thematic analysis in psychology. Qual Res Psychol.

[27] Kuckartz U (2014). Qualitative Text Analysis.

[28] Aronson J (1994). A pragmatic view of thematic analysis. Qual Rep.

[29] Burnard P, Gill P, Stewart K (2008). Analysing and presenting qualitative data. Br Dent J.

[30] Antonovsky A (1996). The salutogenic model as a theory to guide health promotion. Health Promot Int.

[31] Richard L, Gauvin L, Raine K (2011). Ecological models revisited: their uses and evolution in health promotion over two decades. Annu Rev Public Health.

[32] Approach C, Stokols D, Grzywacz JG (2003). Human Environments. Am J Heal Promot.

[33] Michie S, Van Stralen MM, West R (2011). The behaviour change wheel: a new method for characterising and designing behaviour change interventions. Implement Sci.

[34] Leask CF, Harvey JA, Skelton DA (2015). Exploring the context of sedentary behaviour in older adults (what, where, why, when and with whom). Eur Rev Ageing Phys Act.

[35] Owen N, Healy GN, Matthews C E (2010). Too much sitting: the population-health science of sedentary behavior. Ex Sport Sci Revires.

[36] Nicolai S, Benzinger P, Skelton DA (2010). Day-to-day variability of physical activity of older adults living in the community. J Aging Phys Act.

[37] Clemson L, Singh MAF, Bundy A (2012). Integration of balance and strength training into daily life activity to reduce rate of falls in older people (the LiFE study): randomised parallel trial. BMJ.

